# Poorly differentiated thyroid carcinoma arising from a lithium-induced goiter in a patient with schizophrenia: a case report

**DOI:** 10.1186/s13044-021-00115-z

**Published:** 2021-11-19

**Authors:** Jung Ho Choi, Young Ok Hong, Hyo-Jeong Kim, Ah Ra Jung

**Affiliations:** 1 Department of Otorhinolaryngology-Head and Neck Surgery, Nowon Eulji Medical Center, Eulji University School of Medicine, 68 Hangeulbiseok-Ro, Nowon-gu, Seoul, 01830 Republic of Korea; 2 Department of Pathology, Nowon Eulji Medical Center, Eulji University School of Medicine, Seoul, Republic of Korea; 3 Division of Endocrinology and Metabolism, Department of Internal Medicine, Nowon Eulji Medical Center, Eulji University School of Medicine, Seoul, Republic of Korea

**Keywords:** Poorly differentiated thyroid carcinoma, Goiter, Lithium, Schizophrenia

## Abstract

**Background:**

Lithium use causes goiter by increasing serum thyroid-stimulating hormone levels through the inhibition of thyroid hormone release. However, there are no reports of poorly differentiated thyroid carcinoma resulting from lithium-induced goiter. Herein, we report the case of a patient with schizophrenia who developed poorly differentiated thyroid carcinoma arising from a lithium-induced goiter.

**Case presentation:**

A 61-year-old woman who was taking lithium for schizophrenia, visited the thyroid-endocrine center with a 10 × 12 cm anterior neck mass. She had a slowly growing goiter approximately 30 years ago; however, when she came to the hospital for diabetes diagnosis 2 years ago, she had no accompanying symptoms and refused evaluation. Three months before her visit, her dysphagia and dyspnea worsened as the size of her goiter increased rapidly. A neck ultrasound and enhanced thyroid computed tomography (CT) examination revealed a 10.9 × 9.2 × 12.8 cm size multi-lobulated mass on the right thyroid gland, leading to a leftward deviation of the trachea. Diagnostic total thyroidectomy was performed, and microscopic findings and immunohistochemical staining results indicated poorly differentiated thyroid carcinoma (PDTC) in the right thyroid mass. Mutation analyses for *BRAF* and the telomerase reverse transcriptase (*TERT*) promoter was performed. No *BRAF* gene mutations were detected; however, *TERT* promoter C228T point mutation was present in the PDTC. The patient underwent radioactive iodine therapy two months after the surgery. At a recent follow-up 4 months postoperatively, she was taking thyroid hormone replacement and remained in a relatively good health with a serum thyroglobulin level of 0.55 ng/ml.

**Conclusions:**

Thyroid examination of psychiatric patients who develop goiter due to long-term lithium treatment should be monitored regularly, and appropriate investigations and surgery should be performed in a timely manner if the goiter is growing rapidly.

## Background

Thyroid abnormalities are relatively common in patients with schizophrenia, possibly related to underlying genetic link between the disorders and the use of antipsychotic medication for treatment of schizophrenia [1]. The development of goiter has occasionally been observed during lithium treatment in patients with mood-disorders [2, 3]. The initial inhibition of thyroid hormone (TH) release by lithium results in an increase in thyroid-stimulating hormone (TSH) concentration, leading to thyroid enlargement [4]. A recent study reported that Wnt/β-catenin signaling may be relevant in lithium-associated goiters [5, 6]. In vitro studies have shown that lithium significantly increases β-catenin-mediated thyroid follicular cell proliferation, and Wnt/β-catenin signaling plays a crucial role in controlling thyroid follicular cell proliferation [7].

Poorly differentiated thyroid carcinoma (PDTC) is a malignant follicular cell-derived neoplasm of the thyroid, which is an aggressive and rare thyroid carcinoma biologically located between the well-differentiated thyroid carcinoma (WDTC) and anaplastic thyroid carcinoma (ATC) [8]. PDTC is more common in females than in males and in patients over 50 years of age. Furthermore, previously reported genetic alterations in PDTC include the *RAS*, *BRAF*, *TP53*, *PI3KCA*, and *CTNNB1* genes [9, 10]. Most PDTCs occur de novo, and some arise from preexisting, well-differentiated carcinomas of follicular cell origin [11]. Classically, activation of the Wnt pathway in thyroid cancer has been associated with undifferentiated carcinoma, as a second mutational event involved in the progression from a WDTC to a PDTC or ATC and more aggressive thyroid carcinoma [12].

This case report describes a case of diagnosis of PDTC in a patient with schizophrenia due to lack of regular follow-up and appropriate treatment for a huge goiter caused by lithium therapy for decades.

## Case presentation

A 61-year-old woman with diabetes and schizophrenia visited the thyroid-endocrine center at our hospital complaining of dysphagia and repeated vomiting. Furthermore, she experienced increasing dyspnea when lying down from the day before the visit. She was diagnosed with schizophrenia approximately 30 years ago. She had a history of hospitalization in a closed ward when her symptoms were severe, and received antipsychotic medications, including lithium (300 mg/day). She had a slowly growing goiter that began to develop approximately 10 years after taking lithium. She had never undergone any diagnostic tests, including ultrasonography and biopsy, because she had no specific symptoms or discomfort. At the time of admission, the patient’s guardian said that the size of the goiter had dramatically increased in the last 3 months.

**Fig. 1:**
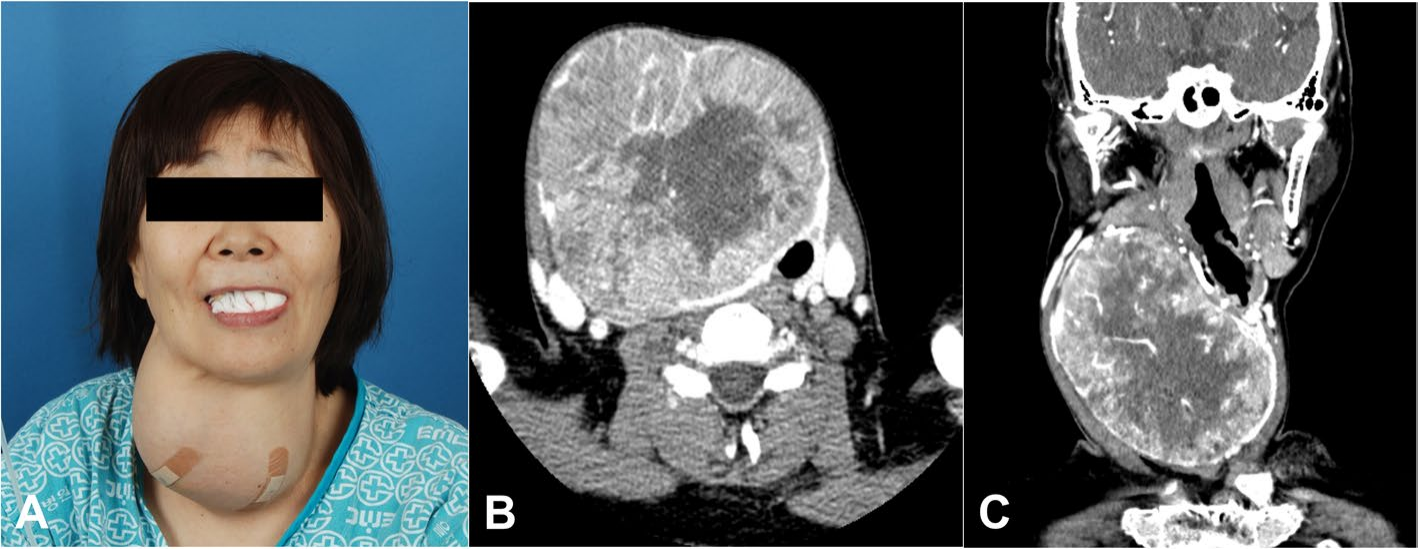
**A** Physical findings. An approximately 10 × 12 cm size fixed and non-tender mass is seen on anterior neck. **B**, **C** Preoperative thyroid CT. It shows a 10.9 × 9.2 cm size lobular shaped mass on the right thyroid gland, and a leftward deviation of the trachea owing to internal calcification and mass

On physical examination, a nontender and hard, huge anterior neck mass of approximately 10 × 12 cm in size was observed. A neck ultrasound and enhanced thyroid computed tomography (CT) examination revealed a multi-lobulated mass measuring 10.9 × 9.2 × 12.8 cm on the right thyroid gland. Moreover, a leftward deviation of the trachea due to internal calcification and mass was noted. Laryngeal examination with fiber optic endoscopy revealed an airway obstruction due to the anterior neck mass, however, the vocal cord mobility was intact (Fig. 1). Laboratory examination showed serum triiodothyronine, free thyroxine, and TSH levels within the normal ranges.

In a multidisciplinary meeting, we discussed the options of whether to perform diagnostic confirmation procedures such as fine needle aspiration biopsy or emergency surgery. We consequently planned total thyroidectomy without additional diagnostic procedures considering the high risk of airway obstruction.

**Fig. 2:**
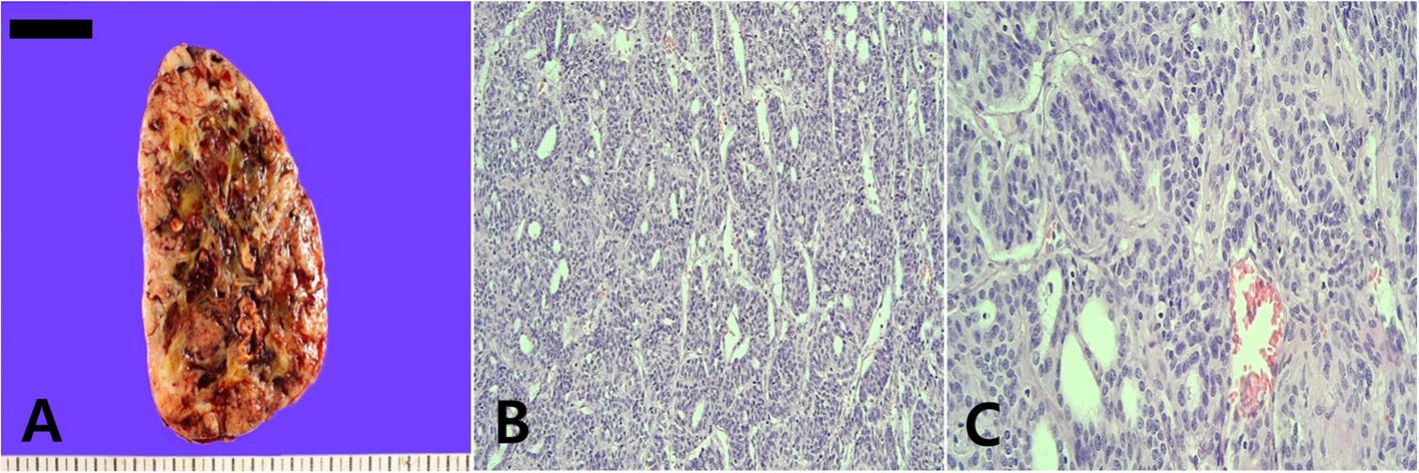
**A** The cut surface of a huge right thyroid mass. **B** The poorly differentiated thyroid carcinoma showed a trabecular, insular, and solid growth pattern (hematoxylin and eosin, × 200). **C** The tumor cells had an increased mitotic count without nuclear features of a papillary thyroid carcinoma (hematoxylin and eosin, × 400)

**Fig. 3:**
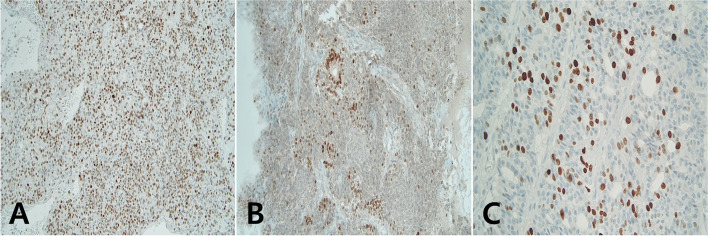
Immunohistochemical stains of the PDTC revealed: **A** Diffuse positivity for p53 immunostaining. **B** Focal positivity for TTF1 immunostaining with loss of poorly differentiated area. **C** Increased Ki-67 labeling index (× 200)

During surgery, an approximately 11.5 × 9 cm mass in the right thyroid gland was removed, and no mass was found in the left thyroid gland. No other extra-thyroidal extensions were noted on gross examination, and recurrent laryngeal nerves were preserved on both sides. Microscopically, the tumor showed a trabecular, insular, and solid growth pattern with necrosis, hemorrhage, and ossification. Additionaly, atypical cells with hyperchromatic convoluted nuclei and increased mitotic figures were observed (Fig. 2). Immunohistochemical staining was performed on formalin-fixed and paraffin-embedded tissues with antibodies against p53, TTF-1, and Ki-67. The tumor cells were diffusely immunopositive for p53, focally immunopositive for TTF-1, and the Ki-67 labeling index was more than 10% in the highest area (Fig. 3). Mutation analyses for *BRAF* and the telomerase reverse transcriptase (*TERT*) promoter were performed. No *BRAF* gene mutations were detected, but the *TERT* promoter C228T point mutation was present in the PDTC.

Approximately 6 h postoperatively, the patient complained of dyspnea and anterior neck swelling in the general ward. Emergency surgery was performed to remove postoperative hematoma and control bleeding. Postoperative ^18^F-FDG PET/CT showed no regional or distant metastases.

The patient received high-dose radioactive iodine therapy (150 mCi) 2 months postoperatively. At a recent follow-up 4 months postoperatively, she was taking TH replacement and remained in a relatively good health with a serum thyroglobulin level of 0.55 ng/ml.

## Discussion

In 1968, Schou et al. first reported an increased occurrence of goiter in patients with bipolar disorder on lithium carbonate treatment [2]. Several studies have reported that the prevalence of goiter in patients receiving lithium ranges from 30 to 59%, and the prevalence is especially high among female patients and patients on the treatment for more than 4 years [4, 7, 13, 14]. In patients with goiter, the thyroid grows approximately twice its normal size, and goiter is generally diffuse; however, nodular goiter has also been reported [15]. Nonetheless, large lithium-induced goiters that cause airway obstruction are rare. Bauer et al. suggested that thyroid enlargement can only be diagnosed reliably by ultrasonography and not by clinical inspection or palpation [13]. In our patient, the goiter was not investigated using ultrasonography or treated for decades; after continuous administration of lithium for schizophrenia, she developed a huge nodular goiter.

The morphology and clinical behavior of PDTC are generally considered intermediate in a tumor progression model of follicular cell-derived thyroid carcinomas, between differentiated thyroid cancer (DTC) and ATC [8]. Most PDTCs arise from well-differentiated carcinomas of follicular cell origin, or are de novo; rarely, they arise from a nodular goiter [11].

The present case describes a heterogeneous cut surface of mass, yellowish tan to brown with necrotic and hemorrhagic areas, and the external surface of the mass was smooth. Microscopically, the tumor was composed of a trabecular, solid, and insular architecture that lacked conventional papillary carcinoma-like nuclear characteristics. Owing to the high mitotic number, hyperchromatic convoluted nuclei, and immunohistochemical panel, a diagnosis of PDTC was made. A follicular carcinoma or papillary carcinoma can be considered for the differential diagnosis of PDTC. A typical follicular carcinoma is encapsulated by a thick fibrous capsule and has a dominant follicular pattern, whereas a papillary carcinoma has characteristic nuclear features, such as intranuclear grooves and pseudoinclusions with a papillary growth pattern [16].

The occurrence of lithium-induced goiter may result from the proliferation of thyrocytes through the activation of tyrosine kinase by lithium ions and its effect on the intracellular signaling associated with the adenylate cycle and Wnt/β-catenin [17]. Classically, activation of the Wnt pathway in thyroid cancer has been related to ATCs, as a second mutational event involved in the progression of poorly or undifferentiated and more aggressive thyroid carcinoma [12]. Wnt signaling activates three different pathways: one canonical or β-catenin-dependent pathway, non-canonical or β-catenin-independent pathway with planar cell polarity pathway, and non-canonical or β-catenin-independent pathway with Ca2+ pathway [12]. In normal thyroid cells, E-cadherin is expressed in the basolateral membrane and its downregulation, by activation of oncogenes such as *BRAF*, has been implicated in the induction of the epithelial mesenchymal transition (EMT) in follicular thyroid cancer cells. E-cadherin keeps β-catenin bound to the cell membrane, and the presence of cytoplasmic β-catenin could be merely a consequence of the loss of E-cadherin expression, although this interaction has not yet been described [18, 19].

*TERT* promoter mutations represent the most common alterations in PDTC, with a stepwise increase from WDTC (9%) to PDTC (40%) and ATC (65 –73%) [20–22]. In the present case, a *TERT* promoter C228T point mutation was detected in the PDTC, suggesting a potential association of genetic factors in the development of PDTC within a lithium-induced goiter.

In conclusion, this case report describes a patient who did not undergo appropriate treatment for huge goiters caused by decades of lithium therapy due to schizophrenia and hypothesizes that the accumulation of long-term gene mutations differentiate goiter into PDTC. Further studies are needed to prove our hypothesis, but patients with goiter on receiving long-term lithium therapy may result in sudden onset of life-threatening symptoms, such as dysphagia and dyspnea. Therefore, we suggest that regular monitoring by performing thyroid examinations, including thyroid ultrasonography and thyroid function tests and appropriate surgical removal are necessary.

## Data Availability

The data that support the findings of this study are available from the Nowon Eulji Medical Center. Data are available from the authors upon reasonable request and with the permission from the Nowon Eulji Medical Center.

## Abbreviations

TH: Thyroid hormone
TSH: Thyroid stimulating hormone
PDTC: Poorly differentiated thyroid carcinoma
WDTC: Well-differentiated thyroid carcinoma
ATC: Plastic carcinoma
CT: Computed tomography
T3: Triiodothyronine
FT4: Free thyroxine
FNAB: Fine-needle aspiration biopsy
RAI: Radioactive iodine
TG: Thyroglobulin
DTC: Differentiated thyroid cancer
EMT: Epithelial mesenchymal transition
